# Genetic Signatures From RNA Sequencing of Pediatric Localized Scleroderma Skin

**DOI:** 10.3389/fped.2021.669116

**Published:** 2021-06-07

**Authors:** Emily Mirizio, Christopher Liu, Qi Yan, Julia Waltermire, Roosha Mandel, Kaila L. Schollaert, Liza Konnikova, Xinjun Wang, Wei Chen, Kathryn S. Torok

**Affiliations:** ^1^Division of Rheumatology, Department of Pediatrics, Children's Hospital of Pittsburgh, University of Pittsburgh, Pittsburgh, PA, United States; ^2^Division of Pediatric Pulmonary Medicine, University of Pittsburgh Medical Center (UPMC) Children's Hospital of Pittsburgh, University of Pittsburgh, Pittsburgh, PA, United States; ^3^Division of Neonatal Medicine, University of Pittsburgh Medical Center (UPMC) Children's Hospital of Pittsburgh, University of Pittsburgh, Pittsburgh, PA, United States; ^4^Clinical and Translational Science Institute, University of Pittsburgh, Pittsburgh, PA, United States

**Keywords:** localized scleroderma, morphea, pediatric rheumatology, bulk RNA sequencing, inflammation

## Abstract

The purpose of this study was to explore the skin transcriptional profile in pediatric localized scleroderma (LS) to provide a better understanding of the altered immune and fibrotic pathways promoting disease. LS is a progressive disease of the skin and underlying tissue that causes significant functional disability and disfigurement, especially in developing children. RNA sequencing (RNAseq) technology allows for improved understanding of relevant cellular expression through transcriptome analysis of phases during LS disease progression (more active/inflammatory vs. inactive/fibrotic) and also permits the use of RNA extracted from existing paraffin-embedded skin tissue, which is important in pediatrics. A strong correlation was observed between the comparison of genes expressed between fresh (RNAlater) and paraffinized skin in healthy and LS subjects, supporting the use of paraffinized tissue. LS gene signatures compared to healthy controls showed a distinct expression of an inflammatory response gene signature (IRGS) composed of IFNγ-, IFNα-, and TNFα-associated genes. GSEA^©^ enrichment analysis showed that the IRGS, including interferon-inducible chemokines such as CXCL9, CXCL10, CXCL11, and IFNγ itself, was more highly expressed in LS patients with more inflammatory lesions. The use of paraffinized skin for sequencing was proven to be an effective substitute for fresh skin by comparing gene expression profiles. The prevalence of the IFNγ signature in the lesion biopsies of active LS patients indicates that these genes reflect clinical activity parameters and may be the promoters of early, inflammatory disease.

## Introduction

**Figure 1:**
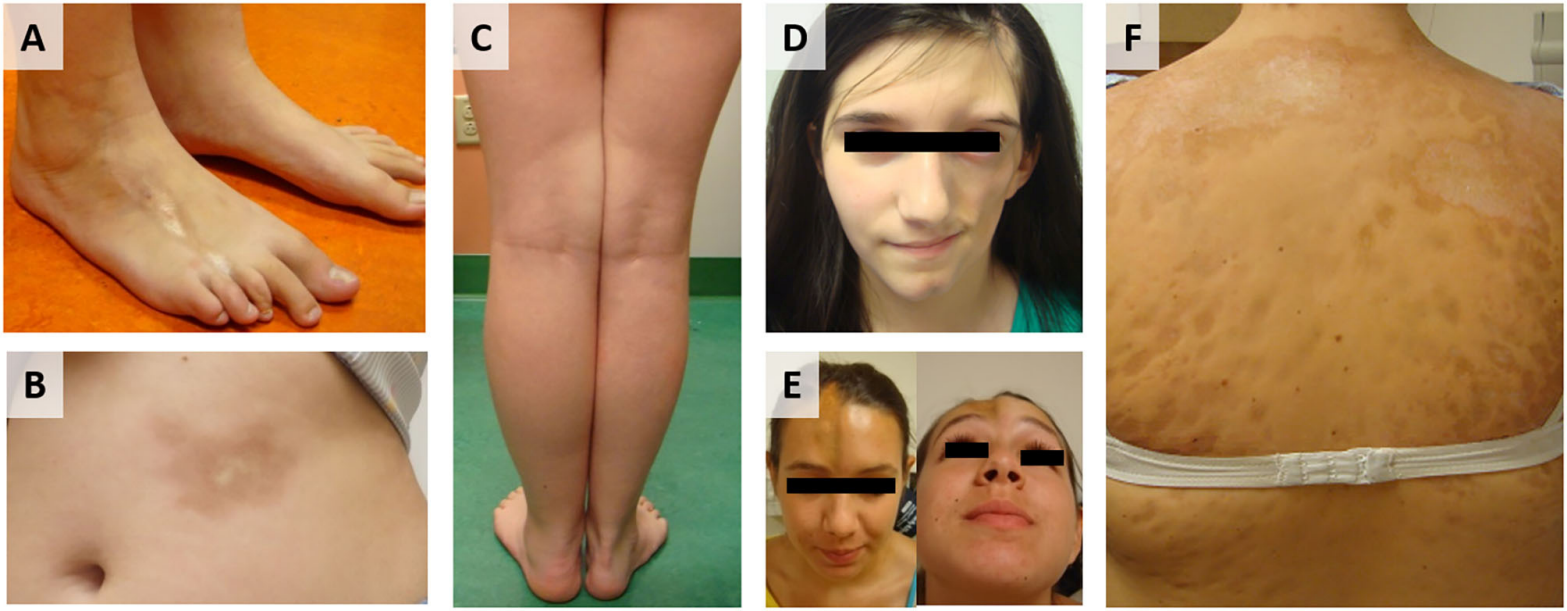
Juvenile Localized scleroderma (LS) subtypes: **(A)** Linear leg lesion with atrophy of the third toe, **(B)** Circumscribed plaque morphea lesion trunk, **(C)** Linear limb lesion with subsequent atrophy and limb length discrepancy, **(D)** LS of the head leading to hemi facial atrophy (Parry Romberg Syndrome), **(E)** LS of the head with en coupe de sabre (ECDS) linear depression forehead/skull, **(F)** Generalized morphea with hyperpigmentation and central sclerosis of lesions.

Localized scleroderma (LS), also known as morphea, is an autoimmune disease characterized by skin fibrosis and subsequent atrophy (typically in bands along the lines of Blaschko) in the absence of vascular and internal-organ involvement, with an annual incidence of 1–3 per 100,000 children (1). LS progression is biphasic, with an inflammatory active phase that is followed by a fibrotic damage phase distinguished by inflammatory infiltrate of the skin, collagen deposition, and subsequent thickening of the deep dermis and subcutis, respectively (2). Clinically, active disease is defined by cutaneous features, such as erythema, skin thickening, new lesions, and lesion expansion, all captured by validated cutaneous clinical outcome measures including the modified Localized Scleroderma Skin Severity Index (mLoSSI) and Physician Global Assessment of Activity (PGA-A) (3–5). Since juvenile LS presents during development (mean age of onset 8 years) and can persist for many years (mean disease duration of 13.5 years) (6), morbidity can be substantial. A recent review of 259 LS patients in the Childhood Arthritis and Rheumatology Research Alliance (CARRA) registry found that 38% had musculoskeletal damage and 25% had limited functional capacity (Figures 1A,C) (7). Damage is most prevalent in linear scleroderma, the pre-dominant juvenile LS subtype (60%) (8, 9).

Biologically, active disease components are still being unraveled. The exact pathogenesis is unclear; however, translational peripheral blood and skin studies in LS support a predominance of CD4+ T cells, macrophages, fibroblasts, and TH1- and IFNγ-associated chemokines/cytokines (10). There is significant elevation of circulating CD4+ IFNγ + T cells (TH1) during active disease (2), along with IFNγ-related proteins CXCL9 [monokine induced by gamma interferon (MIG)] and CXCL10 [interferon gamma-induced protein 10 (IP-10)]. Both CXCL9 and CXCL10 were also present in active LS skin lesions within the perivascular lymphocytic infiltrate of the papillary and reticular dermis. CXCL9 also stained in close approximation to both CD4+TH cells and macrophages (11), suggesting potential interaction between lymphocytes and macrophages via IFNγ chemokine signaling. Overall, these interactions may synergistically promote fibroblasts to increase collagen expression in LS, leading to increased collagen deposition, and fibrosis.

Abrogating the inflammatory response during active LS is critical for limiting disease progression and damage; therefore, further identification of cellular components and molecules involved is paramount. A large-scale, unbiased approach to studying dysregulated IFNγ-mediated pathways and to identify additional pathways involved in LS is now available using large-scale next-generation sequencing (NGS), which is an unbiased method that provides a detailed exploration of dysregulated LS pathways (12). The purpose of this investigation is to further evaluate the LS skin transcriptome using RNA sequencing (RNAseq) to identify up- and downregulated pathways and serve as a platform for future mechanistic studies.

Traditionally, NGS techniques including RNAseq have employed fresh or fresh-frozen (FF) samples rather than formalin-fixed, paraffin-embedded (FFPE) samples due to the potential for degradation, with decreased exonic reads and increased intronic reads with FFPE samples (13). However, more recent studies of other human tissue, including brain, endometrial, lung, and breast cancers (14–22), demonstrated that FFPE samples stored for up to 32 years have been successfully analyzed with RNAseq, introducing the potential for future NGS analysis of widely available archival FFPE samples (23). FFPE samples are more readily available for children who have undergone a diagnostic biopsy, and eliminate the need for a separate research procedure. To our knowledge, skin FFPE vs. FF RNA and sequencing integrity have not been reported. Therefore, our initial goal was to examine the RNA integrity in FFPE skin, followed by RNAseq of LS and healthy FFPE skin specimens to study the differentially expressed genes of the LS transcriptome and their associated pathway(s) analyses.

## Methods

### Clinical Specimens

After obtaining written informed consent, samples for LS subjects were collected through the National Registry for Childhood Onset Scleroderma (NRCOS, IRB #PRO11060222) and healthy controls through IRB #PRO12040127. Accompanying clinical measures and outcome data associated with the subjects' specimens were extracted from these registries. Demographic variables included sex, race, and age at sample visit. Healthy controls were age and sex matched with a 3:1 ratio. Additional clinical variables for LS subjects included LS disease subtype, number of affected body sites, and validated measures of disease activity and severity, which included the Localized Scleroderma Cutaneous Assessment Tool (LoSCAT) and physician global assessments (24, 25). The LoSCAT includes the modified Localized Scleroderma Skin Index (mLoSSI) which quantifies cutaneous disease activity (24). The mLoSSI and the physician global assessment of activity (PGA-A) are the core variables defining disease activity in LS (24) and have been found to be responsive to change (26). The PGA-A is graded on a 100-mm analog scale and includes consideration of the following cutaneous variables: new lesions within the previous month, erythema/violaceous color at the border of the lesion, and skin thickening/induration at the border of the lesion. Patients with mLoSSI > 3 and PGA-A > 10 were considered to have active disease; those with lower scores were considered clinically inactive with a PGA-A and mLoSSI score of 0 (2, 24). Physician documentation of overall judgment of disease state (active/inactive) was also obtained at the study visit.

### RNA Extraction and Sequencing

RNA was extracted from paraffin-embedded skin and a subset of these subjects that had accompanying FF skin collected at the same time which was RNAlater preserved (*n* = 2 LS, *n* = 2 healthy) using the Qiagen AllPrep® DNA/RNA FFPE (Qiagen #80234) and the Qiagen RNeasy Mini (Qiagen #74014) extraction kits, respectively. RNAs were quantified using a Nanodrop ND-100 Spectrophotometer (Nanodrop Technologies, Wilmington, USA) and a 2100 Bioanalyzer (Agilent RNA 6000 Nano Kit, Waldbronn, Germany). Extracted RNA samples were only sequenced if they had a %DV200 (percentage of RNA fragments > 200 nucleotides) (27) >30% for FFPE samples and an RNA integrity number of ≥8 for RNAlater samples to ensure quality control.

Extracted RNA was prepared for sequencing using the Illumina HTS TrueSeq Access library preparation and sequenced on the Illumina NextSeq 500. FastQ files were generated via llumina bcl2fastq2 (Version 2.17.1.14—http://support.illumina.com/downloads/bcl2fastq-conversion-software-v217.html) starting from.bcl files produced by the Illumina NextSeq sequencer. The quality of individual sequences was evaluated using FastQC software (http://www.bioinformatics.babraham.ac.uk/projects/fastqc/).

### RNA and Pathway Analyses

Paired-end RNA sequencing data was aligned using STAR (https://github.com/alexdobin/STAR/releases) and quantified using HTseq (https://htseq.readthedocs.io/en/release_0.11.1/). The human genome reference used for the alignment was hg38—Ensembl Transcripts release 93. Expressed transcripts per sample were evaluated imposing a minimum threshold of five counts per gene to consider it as expressed. Differential expression analysis for all transcripts was performed with the R package DESeq2 (http://bioconductor.org/packages/DESeq2). Genes were analyzed for differences between subject groups using differentially expressed gene (DEG) cutoffs of log2fold change > ±2.5, adjusted p <0.05, counts per gene > 20%, and a false discovery rate (FDR) cutoff of <0.1.

Gene enrichment analysis was performed on significant DEGs between subject groups. Enrichment software (GSEA^©^) with Broad Institute Hallmark gene lists was used to determine whether DEGs between subject groups show statistically significant overrepresentation in a set of genes and any association with disease phenotypes. Gene Ontology (GO) analysis for biological processes, cellular components, and molecular function was also used. PCA and hierarchical clustering of log_2_-transformed fragments-per-kilobase-per-million (FPKM) data were performed using Partek® software. Data was clustered linearly mean centered using Euclidian distance. Color scales were adjusted for presentation purposes. LS clinical subtype data (active/inflammatory vs. stable/disease damage) was applied to these clustering techniques.

### RNAScope® and Immunofluorescent Staining

The location of transcripts of interest from DEG and pathway analyses was analyzed in skin specimens. Formalin-fixed paraffin-embedded biopsies of two LS patients and two healthy controls were used for dual ISH (*in situ* hybridization) and immunofluorescence (IF) multicolor staining. Advanced Cell Diagnostics (ACD) (Newark, CA) RNAscope® LS Multiplex Fluorescent Assay Combined with IF was used. The assay was performed on 3-μm-thick sections using RNAscope® probes targeting CXCL9 (Cat No. 440161), IFNγ (Cat No. 310501-C2), CXCR3 (Cat No. 539251), and CD3 (Cat No. 599391-C2) which were developed by ACD and used according to the manufacturer's recommendations. For IF, primary antibodies against human CD163 as well as appropriate secondary antibodies were used. For multicolor fluorescence microscopy, sections were sequentially stained using the tyramide fluorescence assay. An example is incubation with primary anti-CD163 (clone 56C6; 1:50; Leica MS, Wetzlar, Germany), which was followed by horse anti-rabbit secondary with tyramide Cy5 amplification (PerkinElmer, Waltham, MA). Endogenous peroxidase was blocked with normal horse serum (Sigma-Aldrich, Saint Louis, MO) in Tris-buffered saline (TBS) with bovine serum albumin (BSA). 4′,6′-Diamidino-2-phenylindole (DAPI) counterstaining was performed to visualize the tissue structure. Fluorescence images were recorded using an Echo Revolve fluorescence microscope with filter combinations, specifically DAPI, Cyanine3, and Cy5. The total numbers of RNA and protein-positive cells were counted using ImageJ software (NIH, Bethesda, MD) at ×40 magnification. One slide per sample was stained, and at least three representative images were analyzed per tissue section.

### Statistical Analysis

Sequencing analyses included R package DESeq2 for DEG analyses and GSEA, GO, and Partek® for pathway analyses of DEGs of interest, with cutoffs for significance as mentioned above. Spearman's correlation coefficient analyses were performed to investigate the relationship between gene expression and clinical measures using GraphPad Prism (Version 7.0e, La Jolla, CA, USA). ImageJ software (NIH, Bethesda, MD) was used to count the number of stained transcripts in RNAscope® analyses with % difference between sample type run in GraphPad Prism (Version 7.0e, La Jolla, CA, USA).

## Results

**Figure 2:**
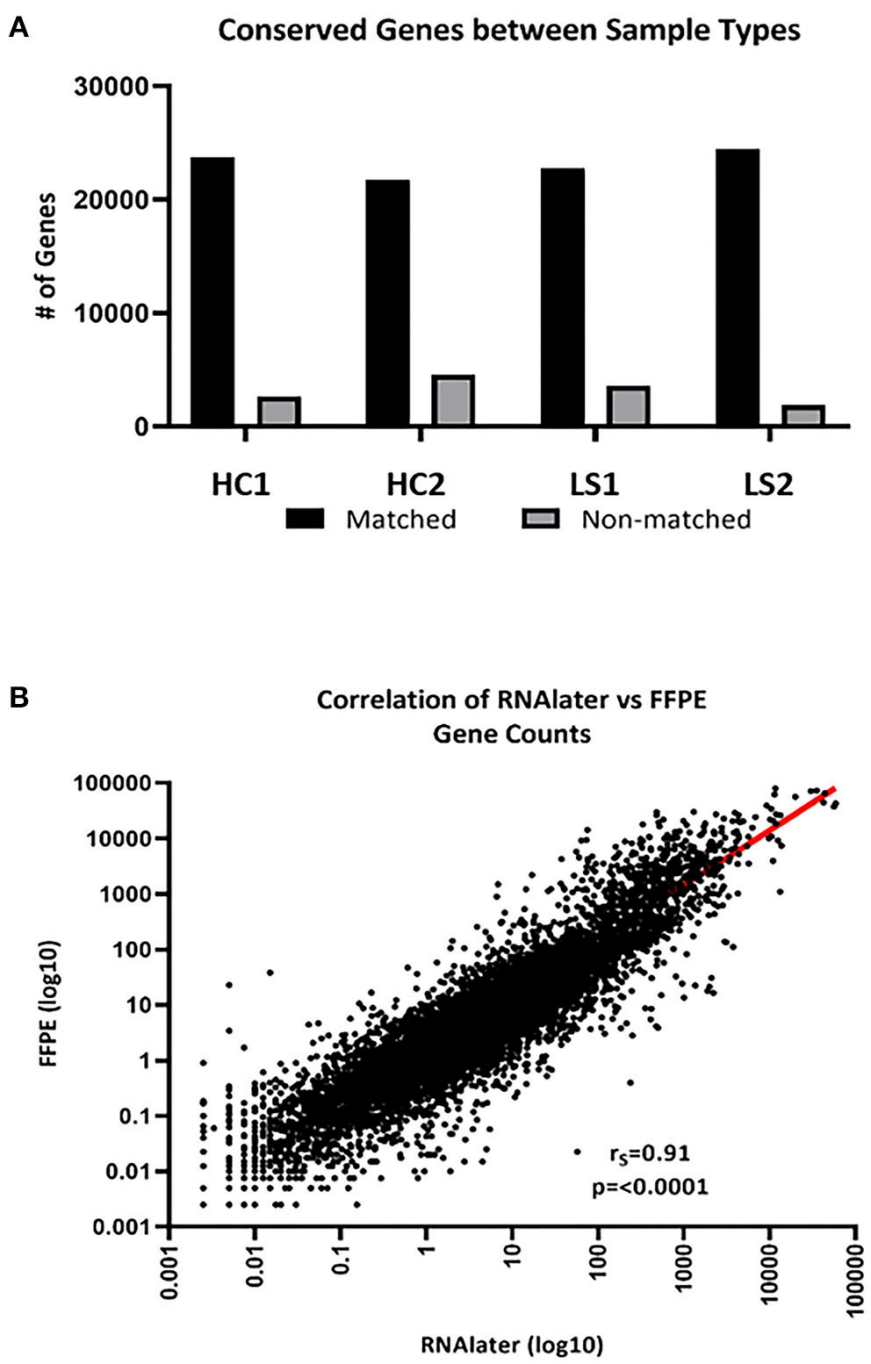
FFPE and FF (RNAlater) generated data sequencing and mapped well per sample type. **(A)** An average of 92% of total genes was conserved between sample types. HC, healthy control and LS, localized scleroderma. **(B)** Of the conserved genes, gene expression correlated significantly (*r*_*s*_ = 0.91, *p* < 0.0001).

The integrity of RNA extracted from FFPE compared to FF skin was compared for paired samples. FastQC data results for RNA extracted from fresh frozen (RNAlater preserved) and paraffinized FFPE skin in healthy (*n* = 2) and LS (*n* = 2) subjects were comparable for measurements of RNA quality, such as total reads and read coverage (Table 1). Once sequenced, FF and FFPE samples were almost indistinguishable and downstream alignment revealed that 92% of mapped genes were conserved between sample types of paired samples (Figure 2A). Gene count data for FF and FFPE paired samples correlated significantly with a correlation coefficient of 0.91 (*p* ≤ 0.0001; Figure 2B). FPKM-normalized data correlated even better with a correlation coefficient of 0.94 (not shown). The high integrity and correlation of expressed genes using FFPE compared to FF-stored skin supported the utilization of FFPE specimens for the RNA Seq analyses of pediatric LS skin compared to healthy controls.

**Table 1:** FastQC analysis results for FFPE and RNAlater sample types support comparable coverage and quality.

	**Total reads (millions)**	**Coverage**	**Avg. coverage depth**	**Avg. quality**	**%GC**
FFPE	39.3 ± 29.1	2.03 ± 0.80	85.5 ± 19.0	35.0 ± 0.13	50.1 ± 3.27
RNAlater	32.9 ± 11.6	2.95 ± 1.56	72.8 ± 35.4	34.2 ± 0.18	52.3 ± 0.42

*FFPE, formalin-fixed, paraffin-embedded; RNAlater, fresh frozen RNA preservative*.

### Pediatric LS vs. Healthy Control Skin Transcriptome

**Figure 3:**
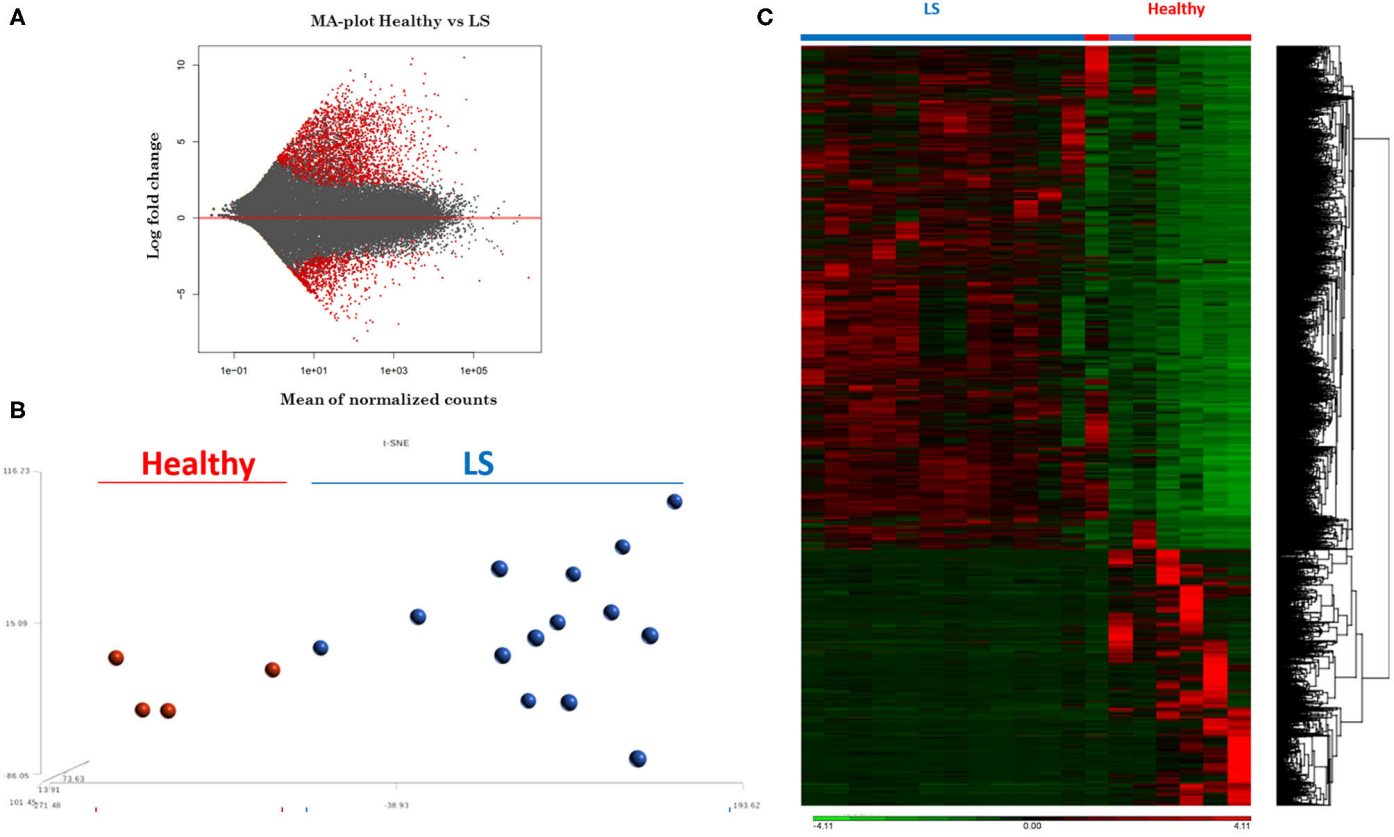
RNAseq expression differences between healthy and localized scleroderma (LS) subject samples. **(A)** Intensity-dependent ratios between two groups of data. Red points denote genes with significant differences. **(B)** tSNE clustering of genetic expression confirms significant differences between the two data groups. **(C)** Heat map showing the overall expression difference between all DEGs comparing LS to healthy samples.

RNA extracted from FFPE samples was analyzed for differentially expressed genes (DEGs) between pediatric LS (*n* = 14 and age-matched healthy (*n* = 4) samples using the R program DESeq. Demographics and clinical variables are provided in Table 2. There were 3,753 DEGs (up and downregulated) between LS and healthy controls, and after applying expression cutoffs 1,302 genes remained significant as demonstrated in the MA plot, a scatterplot of M (log ratio) as the log_2_ fold changes (on the y-axis) vs. the A (mean average) as the mean of normalized counts (on the x-axis) (Figure 3A) (Supplementary Table 1 for full gene list). Visualization of genetic expression per subject using (1) t-Distributed Stochastic Neighbor Embedding (tSNE) clustering techniques (Figure 3B) and (2) heat map expression (Figure 3C) demonstrates that RNA transcript data showed a clear separation between the two groups, with relative homogeneity among LS patients compared to healthy.

**Table 2:** Pediatric healthy control and localized scleroderma (LS) patient demographics and clinical measures.

	**Healthy controls (*n* = 4)**	**LS patients (*****n*** **=** **14)**	
Gender, female, *n* (%)	4 (100)	10 (71)	
Age at time of biopsy (years), mean (SD)	15.5 (1.29)	15.4 (5.16)	
Age at disease onset (years), mean (SD)	–	8.29 (4.38)	
Disease duration (years), mean (SD)	–	4.26 (3.65)	
Ethnicity, *n* (%) (Non-hispanic)	4 (100)	14 (100)	
**Race**, ***n*** **(%)**			
Caucasian	3 (75)	14 (100)	
African American	1 (25)	0	
**Disease subtype**, ***n*** **(%)**			
Linear trunk/limb	–	4 (29)	
Linear face/scalp	–	3 (21)	
Circumscribed morphea	–	3 (21)	
Generalized morphea	–	4 (29)	
Number of affected sites, mean (SD)	–	2.79 (2.86)	
Clinical disease features, median (IQR)	–	Active (*n* = 10)	Inactive (*n* = 4)
mLoSSI^a^	–	6.00 (3.50–6.00)	0.50 (0.00–1.50)
PGA-A^b^	–	37.0 (32.3–44.5)	3.00 (1.00–7.25)

a *mLoSSI: Localized Scleroderma Skin Severity Index*.

b *PGA-A: Physician Global Assessment of Disease Activity*.

GO enrichment and gene set enrichment (GSEA) analysis was performed on DEGs, and GO and GSEA terms with significant enrichment were selected. Significant enrichment groups (*p* ≤ 0.05 and FDR ≤ 0.05) were found to positively relate to TH1- and IFNγ-related immune responses, including the mediation and migration of leukocytes and KRAS signaling (Table 3). Significant groups were also found to negatively relate to DEGs, including the response to the epithelial mesenchymal transition processes (Table 3). Genes encoding for cytokine and inflammatory responses such as CXCL11-, CXCL10-, IL12B-, IFNG-, and IGKV1-related genes were upregulated in disease groups compared to control. Downregulated genes encoding for the epithelial transition stimuli were also found with similar magnitude including PRSS2, WNT5A, and IGFBP2.

**Table 3:** Pediatric localized scleroderma vs. healthy control comparison identifies gene enrichment groups of interest relating to inflammatory and regulatory signatures.

**function**	**Gene symbol**	**Gene description**	**Relative expression**	
**Inflammatory response**			**Log_**2**_FoldChange**	***p*-value**
	CXCL11	C–X–C motif chemokine 11	7.16	0.0000
	IL12B	Interleukin-12 subunit beta: cytokine, defense/immunity protein	5.32	0.0014
	CXCL10	C–X–C motif chemokine 10	3.12	0.0014
	SELE	E-selectin	3.05	0.0000
	MSR1	Macrophage scavenger receptor types I and I: oxidase, receptor, serine protease	2.52	0.0015
**KRAS signaling**				
	IFNG	Interferon gamma	4.24	0.0011
	SLC6A3	Sodium-dependent dopamine transporter: cation transporter	2.91	0.0173
	MAGIX	PDZ domain-containing protein	2.84	0.0017
	KRT1	Keratin, type II cytoskeletal 1	2.67	0.0000
	CPA2	Carboxypeptidase A2: metalloprotease	−7.73	0.0000
**Epithelial–mesenchymal transition**				
	TGM2	Protein-glutamine gamma-glutamyltransferase 2: acyltransferase	−2.47	0.0014
	IGFBP2	Insulin-like growth factor-binding protein 2: protease inhibitor	−2.57	0.0001
	GREM1	Gremlin-1	−2.57	0.0002
	WNT5A	Protein Wnt-5a: signaling molecule	−2.60	0.0004
	CXCL6	C–X–C motif chemokine 6	−3.29	0.0017
	CDH2	Cadherin-2	−3.40	0.0000
	PRSS2	Trypsin-2: serine protease	−6.83	0.0000
**Leukocyte mediation and migration**				
	IGKV1-17	Immunoglobulin kappa variable 1–17	9.88	0.0000
	IGKV2D-29	Immunoglobulin kappa variable 2D−29	9.64	0.0002
	IGHV1-3	Immunoglobulin heavy variable 1–3	8.79	0.0011
	IGKV1-16	Immunoglobulin kappa variable 1–16	8.70	0.0000
	IGKV1D-12	Immunoglobulin kappa variable 1D−12	6.94	0.0000
	SLC7A10	Asc-type amino acid transporter 1	6.74	0.0000
	LEP	Leptin	5.69	0.0000
	IGKV2-24	Immunoglobulin kappa variable 2–24	5.03	0.0000
	CD244	Natural killer cell receptor 2B4: cell adhesion molecule, immunoglobulin receptor superfamily, membrane-bound signaling molecule, protein kinase	4.16	0.0000
	IL36G	Interleukin-36 gamma	3.29	0.0002
	RAET1L	UL16-binding protein 6	3.13	0.0004

Of note was the absence of certain cell type and pathway signatures. Genes relating to certain subsets of T cells that are often connected with autoimmune diseases such as Tregs (FOXP3, CD25) and TH17 (IL-17, RORC) were decreased in gene lists. Additionally, an immune response antagonist, transforming growth factor beta (TGFβ) which has been implicated in promoting activity in other forms of scleroderma, was relatively unaffected in our LS disease samples. While not significantly up or downregulated, the relatively low expression of these genes provides insight in the cells involved in pathogenesis.

### Pediatric Active LS vs. Healthy Control Skin Transcriptome

**Figure 4:**
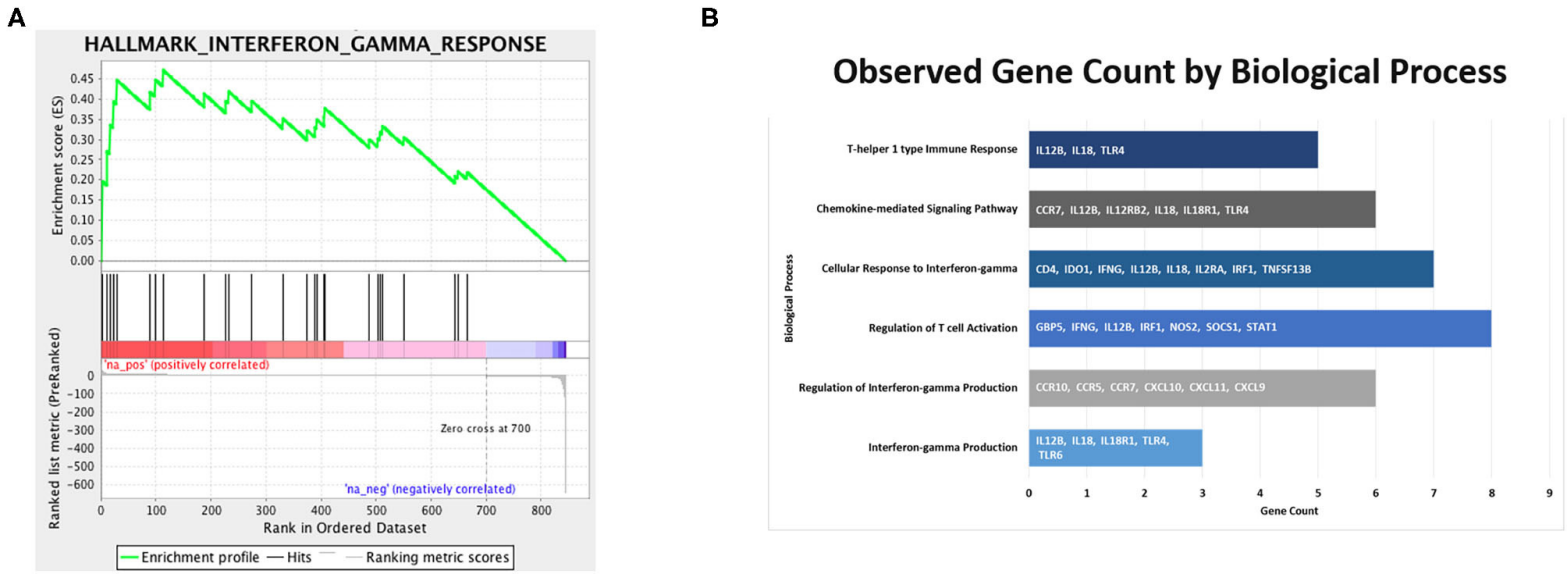
Immune pathways of interest expressed in samples of active localized scleroderma subjects. Significant DEGs were related to known biological, molecular, and cellular processes including T cell migration, activation, and aggregation, and interferon gamma based on GSEA^©^ **(A)** and GO **(B)** enrichment profiles.

Clinical activity, determined by mLoSSI and PGA-A scores, was then used to separate the LS samples into inactive and active groups. Ten active samples and four inactive samples were independently compared to the healthy controls and differential expression calculated. After applying expression cutoffs, 2,366 genes were differentially expressed in the active group compared to controls (see Supplementary Table 2 for a full gene list). Within the active LS subjects, DEG analyses demonstrated a distinct expression of genes encoding for inflammatory responses native to IFNγ/α and TNFα pathways and leukocyte activation and/or regulation, which include CXCL9, CXCL10, CXCL11, IFI27, STAT1, CXCL3, TNF, CSF2, GZMA, and IRF1. Also upregulated in active LS samples were MHC Class II genes, HLA-DQA1 and HLA-DRB1, reflecting activated immune response (Table 4). These pathways were further supported using GSEA^©^ and GO enrichment software (Figures 4A,B). Predominant genes in these GSEA hallmarks were then complied into a master grouping we designated as the “inflammatory response gene signature (IRGS),” composed of 175 genes (see Supplementary Table 3 for full gene list). This gene signature had a high number of genes related to IFNγ that had a high log2 fold change including CXCL11, CXCL10, CXCL9, IRF1, CCL5, CMKLR1, BATF2, OASL, CMPK2, TRIM21, IFI27, GBP4, LAP3, ISG15, XCL1, CD274, GZMA, KLRK1, HLA-DQA1, IDO1, ZBP1, HLA-DRB1, SLAMF7, OAS3, and STAT1, indicating that this signature is highly related to IFNγ reflecting LS literature.

**Table 4:** Pediatric active localized scleroderma (LS) vs. healthy control sample comparison identifies gene enrichment groups of interest relating to inflammatory signatures that was used to develop the Inflammatory Response Gene Signature in LS.

**Function**	**Gene symbol**	**Gene description**	**Relative expression**	
**IFNγ response**			**Log_**2**_FoldChange**	***p*-value**
	CXCL11	C–X–C motif chemokine 11	9.56	0.0015
	XCL1	Lymphotactin	5.60	0.0001
	CXCL10	C–X–C motif chemokine 10	4.65	0.0006
	CD274	Programmed cell death 1 ligand 1: immunoglobulin receptor superfamily!!break Membrane-bound signaling molecule	4.13	0.0003
	OASL	2′-5′-Oligoadenylate synthase-like protein: defense/immunity protein!!break Nucleic acid-binding nucleotidyltransferase	3.98	0.0000
	GZMA	Granzyme A: serine protease	3.83	0.0005
	KLRK1	NKG2-D type II integral membrane protein	3.50	0.0325
	HLA-DQA1	HLA class II histocompatibility antigen, DQ alpha 1 chain	3.26	0.0116
	CCRL2	C–C chemokine receptor-like 2	3.00	0.0161
	CCL5	C–C motif chemokine 5	2.96	0.0015
	TRIM21	E3 ubiquitin-protein ligase TRIM21	2.73	0.0000
	IFI27	Interferon alpha-inducible protein 27, mitochondria	2.71	0.0318
	HLA-DRB1	HLA class II histocompatibility antigen, DRB1-15 beta chain	2.63	0.0012
	SLAMF7	SLAM family member 7: cell adhesion molecule!!break immunoglobulin receptor superfamily!!break Membrane-bound signaling molecule protein kinase	2.61	0.0160
	ISG15	Ubiquitin-like protein ISG15: ribosomal protein	2.53	0.0051
**Cytokine to cytokine receptor signaling**				
	CXCL3	C–X–C motif chemokine 3	9.56	0.0015
	CRLF2	Cytokine receptor-like factor 2: defense/immunity protein	4.65	0.0006
	IL9R	Interleukin-9 receptor: type I cytokine receptor	4.57	0.0004
	TNF	Tumor necrosis factor	4.42	0.0013
	CXCL9	C–X–C motif chemokine 9	3.73	0.0005
	CSF2	Granulocyte-macrophage colony-stimulating factor: cytokine	3.64	0.0000
	IRF1	Interferon regulatory factor 1: nucleic acid binding, winged helix/forkhead transcription factor	3.58	0.0013
	PLA2G2A	Phospholipase A2, membrane associated	3.41	0.0060
	STAT1	Signal transducer and activator of transcription 1-alpha/beta: nucleic acid binding, transcription factor	3.12	0.0000
	FASLG	Tumor necrosis factor ligand superfamily member 6	2.53	0.0001

**Figure 5:**
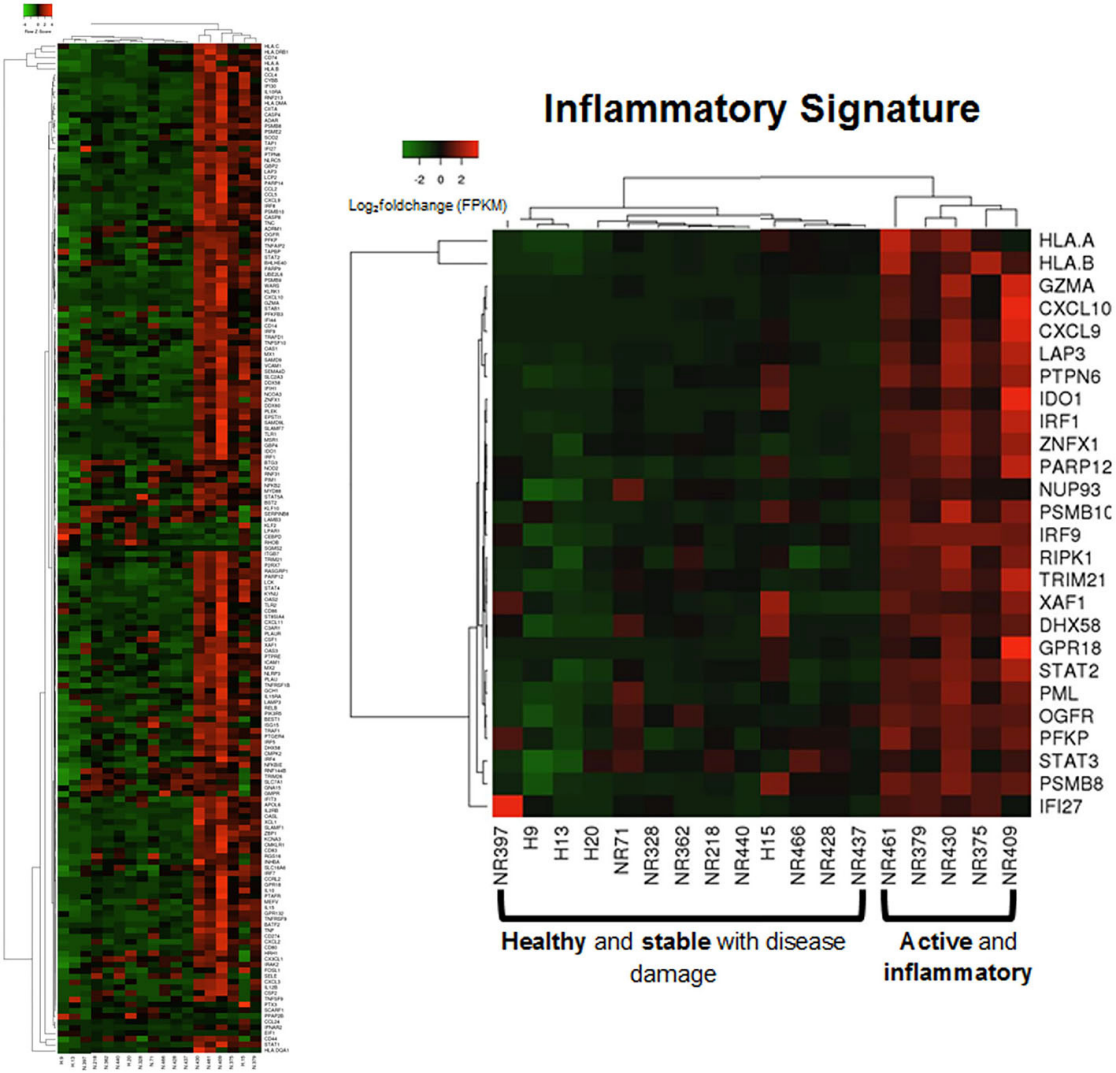
Supervised clustering using IRGS gene set identified a sub-clustering of 5 LS samples with high expression. These samples are considered to be in an active inflammatory disease state (via histological and clinical examination) suggesting the IRGS genes are indicators of active disease (see Supplementary Table 3 for IRGS gene set).

Applying this evaluation of the IRGS gene expression to all LS samples compared to healthy with clinical features, using a cutoff of Spearman's ρ > 0.5 and *p* < 0.05, 57 of a total of 175 genes within the inflammatory signature group (ISRG) correlated strongly and positively with validated clinical disease activity measures, the mLoSSI and the PGA-A. Several interferon pathway-associated genes (inducible, regulatory, and receptor) and cytokine signaling genes were highly correlated, most with both measures. Notable were IFI44, IFT3, IRF5, IL-15RA, CXCL2, CD86, and STAT4 (Table 5). Applying hierarchal clustering of the genes included in the IRGS (Cluster 3.0 and Java TreeView) demonstrated that five extremely clinically active individuals out of the 14 total LS patient samples clustered together with an upregulated expression of this inflammatory signature (Figure 5). These subjects were composed of different clinically defined LS subtypes (two linear extremity, two circumscribed, one generalized morphea).

**Table 5:** Gene expression (FPKM) correlations with clinical parameters of disease activity.

**Clinical parameter**	**Gene symbol**	**Gene description**	**Correlation metrics**	
**mLoSSI**			**Spearman's Rho**	***p*-value**
	IFI44	Interferon-induced protein 44	0.78	0.0017
	CASP8	Caspase-8: cysteine protease, protease inhibitor	0.76	0.0024
	IL15RA	Interleukin-15 receptor subunit alpha	0.74	0.0037
	IFNAR2	Interferon alpha/beta receptor 2: defense/immunity protein, type I cytokine receptor, type II cytokine receptor	0.70	0.0071
	IFIT3	Interferon-induced protein with tetratricopeptide repeats 3	0.69	0.0076
	IRF5	Interferon regulatory factor 5	0.69	0.0085
	KLRK1	NKG2-D type II integral membrane protein	0.69	0.0085
	CXCL2	C–C chemokine receptor-like 2	0.69	0.0085
	KYNU	Kynureninase, hydrolase	0.67	0.0106
	CD86	T-lymphocyte activation antigen CD86	0.67	0.0109
	PARP14	Protein mono-ADP-ribosyltransferase PARP14	0.67	0.0113
	XAF1	XIAP-associated factor 1	0.65	0.0135
	MSR1	Macrophage scavenger receptor types I and II: oxidase, receptor, serine protease	0.65	0.0139
	RNF213	E3 ubiquitin-protein ligase RNF213	0.64	0.0152
	SAMD9	Sterile alpha motif domain-containing protein 9	0.64	0.0157
	PLEK	Pleckstrin, cytoskeletal protein	0.64	0.0161
	P2RX7	P2X purinoceptor 7, ligand-gated ion channel	0.64	0.0166
	NLRP3	NACHT, LRR, and PYD domain-containing protein 3	0.62	0.0202
	DDX60	Probable ATP-dependent RNA helicase DHX60	0.61	0.0218
	TLR2	Toll-like receptor 2	0.61	0.0236
	SEMA4D	Semaphorin-4D, membrane-bound signaling molecule	0.61	0.0243
	ST8SIA4	CMP-N-acetylneuraminate-poly-alpha-2,8-sialyltransferase	0.61	0.0243
	STAT4	Signal transducer and activator of transcription 4	0.61	0.0243
	PLAU	Urokinase-type plasminogen activator, serine protease	0.60	0.0249
	IL10RA	Interleukin-10 receptor subunit alpha	0.60	0.0249
	SLC16A6	Monocarboxylate transporter 7	0.60	0.0262
	RASGRP1	RAS guanyl-releasing protein 1	0.60	0.0262
	DDX58	Probable ATP-dependent RNA helicase DHX58	0.59	0.0276
	ADAR	Double-stranded RNA-specific adenosine deaminase	0.59	0.0283
	CCRL2	C–C chemokine receptor-like 2	0.59	0.0290
	IRAK2	Interleukin-1 receptor-associated kinase-like 2	0.59	0.0297
	MEFV	Pyrin, ubiquitin-protein ligase	0.59	0.0297
	MX1	Interferon-induced GTP-binding protein Mx1	0.59	0.0297
	MX2	Interferon-induced GTP-binding protein Mx2	0.59	0.0304
	PFKFB3	6-phosphofructo-2-kinase/fructose-2,6-bisphosphatase 3	0.58	0.0312
	TNFRSF9	Tumor necrosis factor receptor superfamily member 9	0.58	0.0313
	OAS3	2′-5′-Oligoadenylate synthase 3	0.57	0.0343
	LAMP3	Lysosome-associated membrane glycoprotein 3: membrane trafficking regulatory protein	0.57	0.0360
	CD44	CD44 antigen, transmembrane signal receptor	0.57	0.0369
	LCP2	Lymphocyte cytosolic protein 2, scaffold/adaptor protein	0.56	0.0386
	CXCL3	C–X–C motif chemokine 3	0.56	0.0394
	CMKLR1	Chemokine-like receptor 1	0.56	0.0395
	TNFRSF10	Tumor necrosis factor receptor superfamily member 10	0.55	0.0433
	OGFR	Opioid growth factor receptor, transmembrane signal receptor	0.55	0.0433
	PARP12	Protein mono-ADP-ribosyltransferase PARP12	0.55	0.0433
	APOL6	Apolipoprotein L6	0.55	0.0463
	C3AR1	C3a anaphylatoxin chemotactic receptor	0.54	0.0473
	OAS2	2′-5′-Oligoadenylate synthase 2	0.54	0.0473
	TLR1	Toll-like receptor 1	0.54	0.0489
**PGA-A**				
	CASP8	Caspase-8: cysteine protease, protease inhibitor	0.71	0.0063
	IL15RA	Interleukin-15 receptor subunit alpha	0.70	0.0063
	KLRK1	NKG2-D type II integral membrane protein	0.61	0.0225
	IFI44	Interferon-induced protein 44	0.58	0.0320
	MSR1	Macrophage scavenger receptor types I and II: oxidase, receptor, serine protease	0.57	0.0342
	IFIT3	Interferon-induced protein with tetratricopeptide repeats 3	0.57	0.0351
	IFNAR2	Interferon alpha/beta receptor 2: defense/immunity protein, type I cytokine receptor, type II cytokine receptor	0.57	0.0351
	IRF5	Interferon regulatory factor 5	0.57	0.0368
	ST8SIA4	CMP-N-acetylneuraminate-poly-alpha-2,8-sialyltransferase	0.57	0.0368
	TNFRSF9	Tumor necrosis factor receptor superfamily member 9	0.56	0.0400
	KYNU	Kynureninase, hydrolase	0.55	0.0422

*Strong correlation between interferon genes within the Inflammatory Response Gene Signature and clinical disease activity measures*.

*mLoSSI, Localized Scleroderma Skin Severity Index*.

*PGA-A, Physician Global Assessment of Disease Activity*.

**Figure 6:**
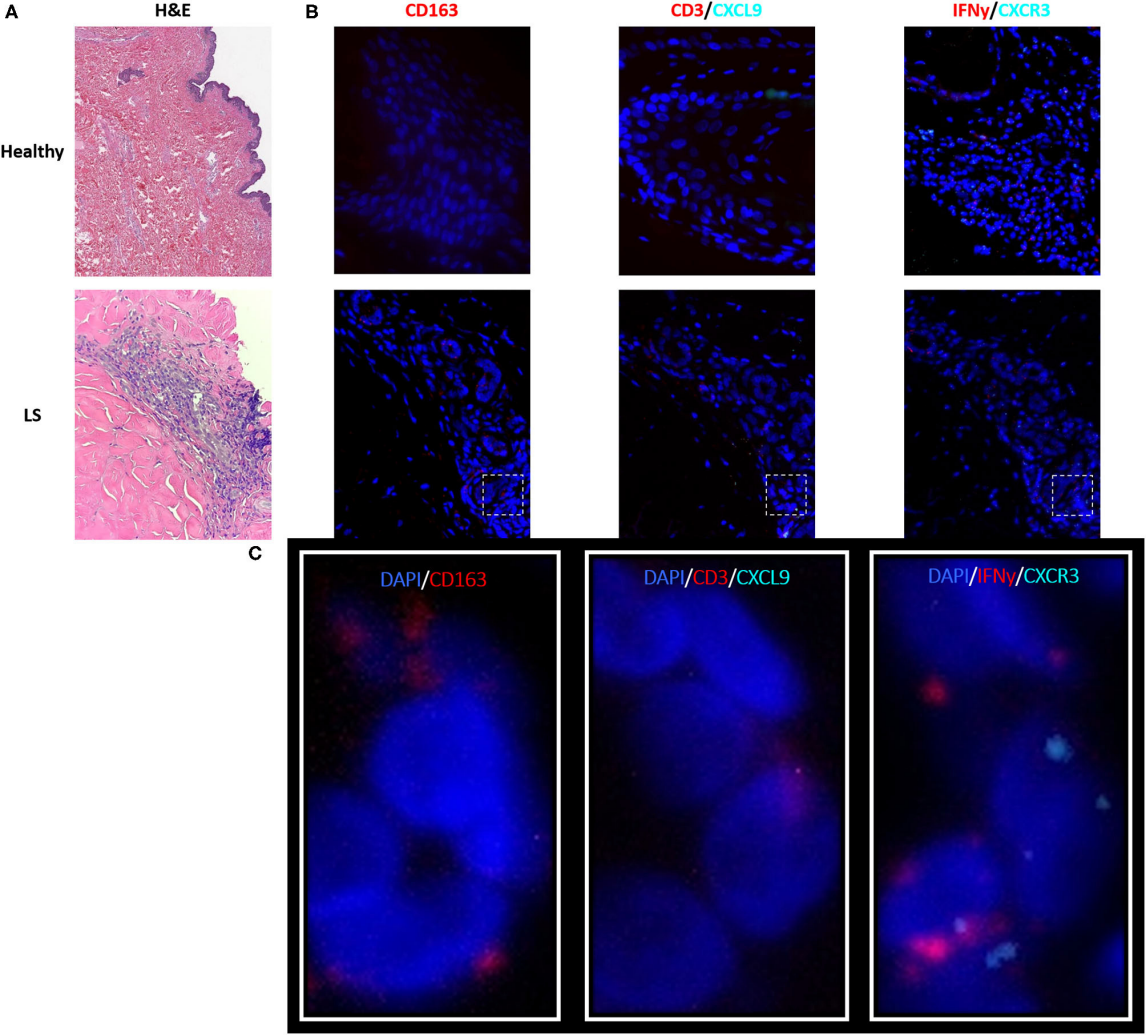
Visualization in the skin identifies T cell and macrophage co-localization, with CXCL9+ and IFNγ+ macrophages adjacent to CXCR3+ T cells in localized scleroderma (LS) skin. **(A)** H&E demonstrates inflammatory infiltrate extending into the lower reticular dermis in LS while the healthy sample demonstrates basal levels of minimal inflammatory cells that are typically localized to periadnexal structures. **(B)** RNAscope® fluorescent multiplex imaging showed increased macrophages (CD163+), CXCL9, and IFNy transcript in LS skin as compared to controls. **(C)** Macrophage-specific (CD163+) CXCL9 and IFNγ staining in close approximation to CXCR3+T cells.

As described in the literature, IFNγ-related protein, CXCL9, had an upregulated transcriptional expression specifically in patients with active disease. Dual RNA and protein staining of LS and healthy negative controls showed localization of CXCL9 expression on CD163+ macrophages (Figure 6). Increased macrophage infiltration, with increased CXCL9 and IFNγ expression, was observed in LS tissue as compared to healthy tissue, especially in areas of inflammation when compared to H&E (Table 6). Macrophage-specific (CD163+) CXCL9 and IFNγ cells stained in close approximation to CXCR3+ T cells.

**Table 6:** Immunofluorescent and RNAscope® fluorescent multiplex imaging of localized scleroderma and control skin.

	**Healthy (H) %**	**Localized scleroderma (LS) %**	**Difference (LS vs. H) %**
**RNA Scope expression relative to total cell count**			
CXCR3	3.11	5.31	2.20
IFNγ	5.34	22.81	17.47
CXCL9	5.31	9.62	4.30
CD3	7.81	10.74	2.92
CD163	0.00	13.36	13.36

*The number of positively stained cells per total number of cells per field at 40 × was used to compare abundance of transcript staining in macrophages and T cells*.

### Pediatric Inactive LS vs. Healthy Control Skin Transcriptome

The remaining inactive samples were then investigated. After applying expression cutoffs, 2,247 genes were differentially expressed in the inactive group (see Supplementary Table 4 for full gene list). Genes encoding for ECM formation and dermal restructuring such as development and differentiation of the epidermis, ECM organization, and keratinization were the most significant after GO and GSEA enrichment analysis (Table 7). The genes included in these enrichment groups included COL17A1, KRT173, FLG, and COL17A. Additionally, transcription factors that are important in regulating the production of factors that control epithelial–mesenchymal interactions, cellular proliferation, and extracellular matrix production such as WNT, ERK, PI3K-TBX, FOX, RUNX, and SRF were demonstrated to be highly expressed. The inactive sample DEGs contain a much higher prevalence of collagen and keratin genes with significantly fewer genes relating to the IGRS inflammatory signature.

**Table 7:** Pediatric inactive localized scleroderma vs. healthy control sample comparison identifies gene enrichment groups of interest related to fibrotic signatures.

**Function**	**Gene symbol**	**Gene description**	**Relative expression**	
**Epidermis development and differentiation**			**Log_**2**_FoldChange**	***p*-value**
	KRT73	Keratin, type II cytoskeletal 73	5.25	0.0049
	KRT2	Keratin, type II cytoskeletal 2 epidermal	4.65	0.0440
	HES5	Transcription factor HES-5: basic helix-loop-helix transcription factor	4.51	0.0077
	LCE1B	Late cornified envelope protein 1B	4.25	0.0036
	FLG	Filaggrin: cytoskeletal protein	4.23	0.0219
	KLK12	Kallikrein-12: serine protease	3.99	0.0033
	LCE1A	Late cornified envelope protein 1A	3.89	0.0040
	DCT	L-Dopachrome tautomerase: oxidase, oxygenase	3.79	0.0018
	CALML5	Calmodulin-like protein 5	3.77	0.0101
	PDZD7	PDZ domain-containing protein 7	3.55	0.0094
	SOX21	SOX-21: HMG box transcription factor	3.46	0.0010
	RBP2	E3 SUMO-protein ligase: G-protein modulator	3.26	0.0430
	EVPL	Envoplakin: intermediate filament binding protein	3.24	0.0001
	C1orf68	Skin-specific protein 32	3.22	0.0537
	KRT12	Keratin, type I cytoskeletal 12	3.22	0.0288
	KRT1	Keratin, type II cytoskeletal 1	3.22	0.0046
	POU3F1	POU domain, class 3, transcription factor 1	3.06	0.0009
	COL7A1	Collagen alpha-1(VII) chain	3.05	0.0002
	CASP14	Caspase-14: cysteine protease, protease inhibitor	3.05	0.0088
	PKP1	Plakophilin-1: intermediate filament binding protein	3.00	0.0006
	GRHL3	Grainyhead-like protein 3 homolog: transcription factor	2.98	0.0008
	EDAR	Tumor necrosis factor receptor superfamily member EDAR	2.90	0.0343
	LOR	Loricrin	2.88	0.0119
	COL17A1	Collagen alpha-1(XVII) chain	2.85	0.0016
	CDH3	Cadherin-3	2.80	0.0003
	LCE2B	Late cornified envelope protein 2B	2.77	0.0111
	TGM1	Protein-glutamine gamma-glutamyltransferase K: acyltransferase	2.72	0.0003
	KRT10	Keratin, type I cytoskeletal 10	2.71	0.0105
	SFN	14-3-3 protein sigma: chaperone	2.71	0.0007
**ECM organization**				
	PLG	Plasminogen: serine protease	−2.97	0.0313
	PRSS1	Trypsin-1: serine protease	−4.38	0.0308
	CTRB1	Chymotrypsinogen B: serine protease	−6.90	0.0008
	CTRB2	Chymotrypsinogen B2: serine protease	−7.09	0.0412
	FGA	Fibrinogen alpha chain	−8.15	0.0457

### Pediatric Active LS vs. Inactive LS Skin Transcriptome

The 10 active and four inactive samples were then compared to each other using differential gene analysis. Due to sample similarity, the magnitude of expression was decreased in this comparison so an adjusted cutoff to log_2_fold change > ±0.5 was used. After applying adjusted expression cutoffs, 1,213 genes were differentially expressed in the active group compared to the inactive group (see Supplementary Table 5 for full gene list). The enrichment groups discovered after GO and GSEA enrichment analysis were much broader in category, including epithelial–mesenchymal transition. Enrichment groups specifically related to immune or functional pathways including genes encoding for IL2 STAT5, IL6 JAK/STAT3, TNFα via NFKB, and KRAS signaling were the most significant (Table 8). The genes included in these enrichment groups included IL1B, NFKBIA, DUSP1, and JUNB. A positive correlation of some of the genes included in the enrichment groups, such as IFI44, CASP8, IFNAR2, IL15RA, and IFNAR2, among others, with disease activity scores of mLoSSI and PGA-A was seen (Table 5).

**Table 8:** Pediatric active vs. inactive localized scleroderma sample comparison identifies gene enrichment groups of interest.

**Function**	**Gene symbol**	**Gene description**	**Relative expression**	
**Epithelial–mesenchymal transition**			**Log_**2**_FoldChange**	***p*-value**
	CALD1	Caldesmon 1	1.06	0.0025
	FSTL1	Follistatin Like 1	1.04	0.0021
	CDH2	Cadherin 2	1.02	0.0037
	FBN2	Fibrillin 2	0.90	0.0006
	SPARC	Secreted protein acidic and cysteine-rich protein coding	0.85	0.0024
	ANPEP	Alanyl aminopeptidase, membrane	0.85	0.0061
	ITGB3	Integrin subunit beta 3	0.83	0.0185
	MYL9	Myosin light chain 9 protein coding	0.82	0.0185
	ITGB5	Integrin subunit beta 5	0.81	0.0031
**IL2 STAT5 signaling**				
	SH3BGRL2	SH3 domain-binding glutamic acid-rich-like protein 2	1.11	0.0002
	GATA1	Erythroid transcription factor	0.96	0.0021
	SELP	P-selectin	0.88	0.0005
	FAH	Fumarylacetoacetase	0.86	0.0015
	SLC2A3	Solute carrier family 2, facilitated glucose transporter member 3	0.83	0.0021
	IGF1R	Insulin-like growth factor 1 receptor	0.81	0.0004
	PIM1	Serine/threonine-protein kinase pim-1	0.75	0.0003
**IL6 JAK/STAT3 signaling**				
	CBL	E3 ubiquitin-protein ligase CBL	0.87	0.0009
	PF4	Platelet factor 4, chemokine	0.85	0.0015
	ITGB3	Integrin beta-3	0.83	0.0185
	IL1R2	Interleukin-1 receptor type 2	0.80	0.0074
	PIM1	Serine/threonine-protein kinase pim-1	0.75	0.0003
	IL17RA	Interleukin-17 receptor A	0.71	0.0002
	ACVRL1	Serine/threonine-protein kinase receptor R3	0.68	0.0310
	IL1B	Interleukin-1 beta	0.65	0.0033
	IFNGR2	Interferon gamma receptor 2	0.65	0.0014
**KRAS signaling**				
	TMEM158	Transmembrane protein 158	1.06	0.0026
	F13A1	Coagulation factor XIII A chain	1.00	0.0002
	BPGM	Bisphosphoglycerate mutase	0.91	0.0014
	GYPC	Glycophorin-C	0.89	0.0049
	CBL	E3 ubiquitin-protein ligase CBL	0.87	0.0009
	PLEK2	Pleckstrin-2, cytoskeleton	0.83	0.0113
	TFPI	Tissue factor pathway inhibitor	0.81	0.0062
	PLVAP	Plasmalemma vesicle-associated protein	0.77	0.0236
	PTGS2	Prostaglandin G/H synthase 2	0.76	0.0018
**TNFα** **signaling via NFKB**				
	PFKFB3	6-Phosphofructo-2-kinase/fructose-2,6-bisphosphatase 3	0.92	0.0011
	BCL6	B-cell lymphoma 6 protein	0.81	0.0007
	TNFRSF9	Tumor necrosis factor receptor superfamily member 9	0.70	0.0025
	BCL3	B-cell lymphoma 3 protein	0.67	0.0029
	IRS2	Insulin receptor substrate 2	0.65	0.0184
	IL1B	Interleukin-1 beta	0.65	0.0033
	IFNGR2	Interferon gamma receptor 2	0.65	0.0014
	PTPRE	Receptor-type tyrosine-protein phosphatase epsilon	0.64	0.0001
	FOSL2	FOS Like 2, AP-1 Transcription Factor Subunit	0.63	0.0010

### Subanalysis of Juvenile LS Disease Subtypes

**Figure 7:**
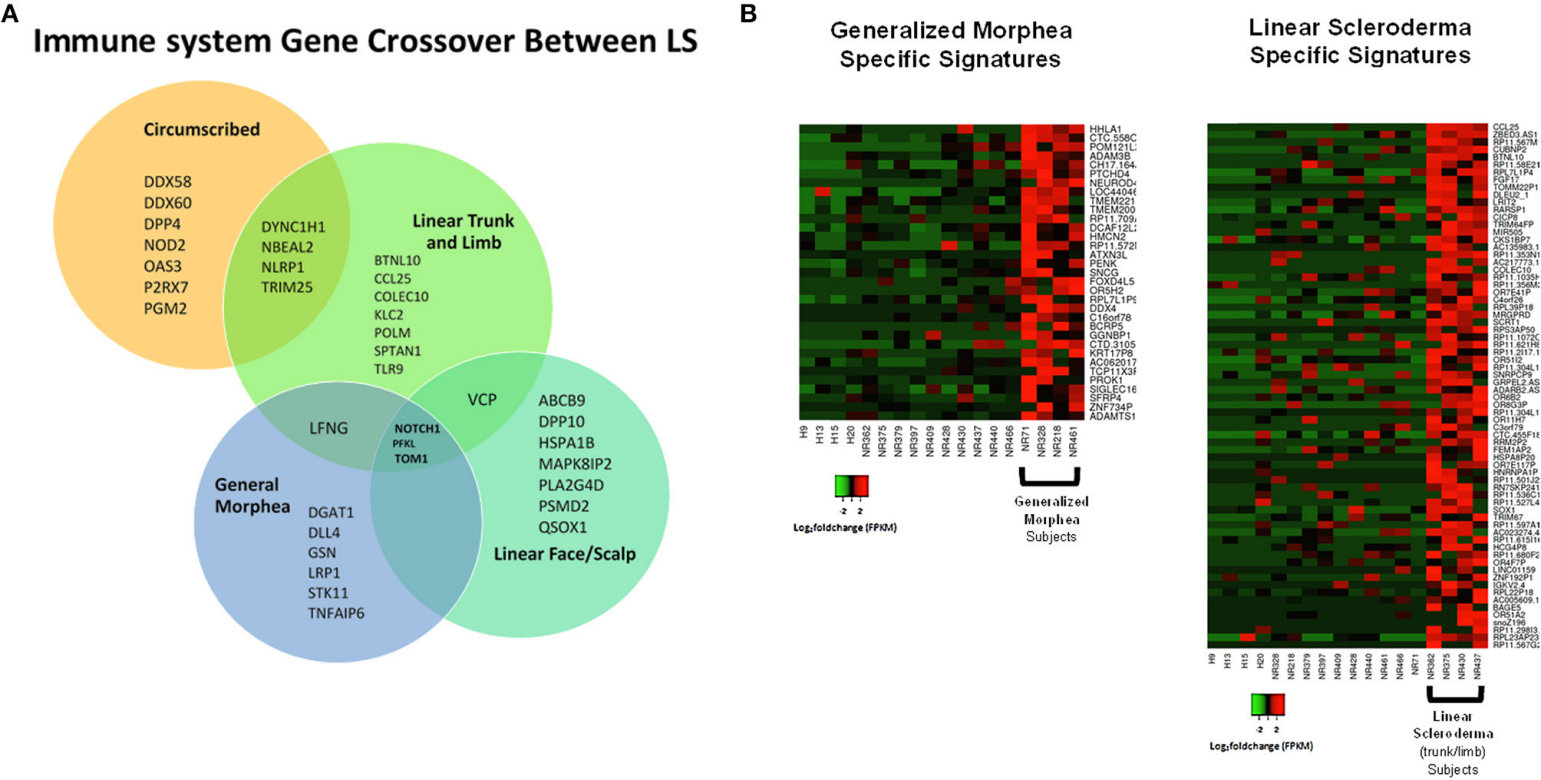
Shared and divergent immunological pathways among localized scleroderma subtypes. **(A)** Venn diagram demonstrates genetic immunological overlap occurring between subtypes, while having some uniquely different gene expression. **(B)** The two main LS subtypes, generalized morphea and linear scleroderma, display some areas of unique gene expression profiles that correspond to unique enrichment groups.

Samples were then separated based on clinical subtype of disease (Figure 1), which included four linear scleroderma of the trunk and limb, three linear scleroderma of the face and scalp, three circumscribed morphea, and four generalized morphea samples, and then compared to healthy controls independently. For each comparison, around 50 genes were differentially expressed. Of these DEGs, many overlapped between subtypes with some specific expression for each group (Figure 7A). Clustering of all samples showed that even within LS, distinct subgroupings occurred. While many genes were common compared to healthy controls, each subtype has unique genetic traits correlating to cutaneous disease manifestations and different immune profiles (Figure 7A). Enrichment of subtype-specific DEGs showed that generalized morphea and circumscribed morphea subtypes (more common in adult-onset) had increased activity to the inflammasome pathway in cellular response categories (GSEA), but only generalized morphea had elevated TNF-related molecular adhesion and migration activity (GSEA) as well as T-cell activator differentiation (GO) (Figure 7B). Circumscribed morphea had elevated interferon and ubiquitin-related cell activation pathways in addition to seven inflammatory activation genetic profiles. Linear scleroderma (more common in pediatric onset) had increased activity of the immune activation-related genetic profile and high expression of cell killing, immune response, and chemical synapse pathways (GO) (Figure 7B). Linear scleroderma of the face and scalp had increased activity of the neural inflammatory signaling pathways (GSEA), which may correspond to the brain lesions associated with this subtype (28). Linear scleroderma of the trunk and limb subtype shared the most genes with the other subtypes, especially circumscribed, and individually had increased collagen deposition in the intrinsic prothrombin activation pathway (GSEA). An important limitation of this subtype subanalysis is the low sample number and an unequal distribution of active and inactive samples within each subtype group.

## Discussion

Gene expression profiling has traditionally used RNA extracted from fresh frozen (FF) tissue that is typically flash frozen or frozen in RNA stabilization solution such as RNAlater® (14). Unfortunately, there are many difficulties associated with the collection of FF specimens mostly pertaining to availability of enough tissue (29). However, abundant archival FFPE samples, retained from diagnostic histopathology procedures, offer an alternative to the scarcer FF sample (30). In pediatric research, the use of paraffin blocks stored from diagnostic procedures also reduces the need for children to undergo additional research biopsies. The potential for gene expression analysis lying within FFPE archives presents researchers with the opportunity to pursue retrospective studies focusing on correlations between gene expression patterns and disease states or phenotypes (31). As the storage of biopsy samples customarily involves formalin fixation and paraffin embedding, the optimization of methods for performing gene expression analysis on FFPE tissues will significantly expand the preexisting tissue resources from which researchers can draw data (18). Already, studies have demonstrated that FFPE samples may serve as a comparable alternative to FF samples for gene expression analysis in glioblastoma, endometrial, lung, and breast cancer tissues (18–22). In 2014, Hedegaard et al. investigated the potential applications of RNA-Seq and DNA Exome-Seq procedures on FFPE samples of colon, prostate, and bladder carcinomas, ultimately finding that after mapping analysis, clear correlations and similar sequence variants existed between RNA-Seq and DNA Exome-Seq results, respectively (19). Iddawella et al. performed correlational analysis on RNA profiles of matched FF and FFPE breast cancer samples and found correlations ranging from 0.83 to 0.89 which improved to 0.96 to 0.98 upon gene selection, thus solidifying that FFPE tissues could serve as reliable sources for expression profiling (32).

Sequencing of paired RNAlater and FFPE pediatric skin tissue samples has not been performed to date, and our data being presented yielded comparable results in quality and mapped genes between healthy and disease (LS) pediatric skin, with correlation analyses of RNA profiles of matched samples similar to those recently reported in breast cancer and other tissue sources (18–21, 32, 33). A high correlation of RNAlater and FFPE tissue found in this investigation indicates that FFPE tissue can be utilized for bulk RNA-seq in place of fresh or FF tissue in both healthy and disease state of the skin. This may open the door for several autoimmune and other skin diseases to be able to take advantage of stored tissue, especially in pediatrics in which repeat fresh biopsies are not tolerated well. These results supported our decision to use FFPE skin samples for RNA sequencing of 14 additional skin samples to evaluate the difference in transcriptomic expression between LS and healthy controls.

### Unique Transcriptomic Findings in Pediatric Localized Scleroderma

To date, studies examining the pathogenesis of LS have consisted of reports of circulating chemokine profiles or antibodies, flow cytometry of peripheral blood, and immunostaining often in a limited number of samples or without controls (34–37). Most of these studies have observed increased serum CXCL9 and CXCL10 levels that are associated with increased clinical measures of disease activity (2, 11, 38–41). Despite these studies, the pathogenesis of LS is largely unknown with transcriptional profiling by microarray or RNA bulk sequencing limited to two available studies.

Little is known about the transcriptional profile of localized scleroderma (LS) and even less about pediatric LS. Only a few studies have investigated the gene expression of LS through NGS methods, and our study is the first to examine a wide range of subtypes from pediatric LS subjects. In SSc, transcriptional investigation has been successful at classifying samples based on genetic differences that could indicate or predict disease progression and potential future treatment plans. In the landmark SSc microarray skin expression studies defining these classifications by Milano et al. there were three adult LS subjects included with the 24 SSc subjects. The skin expression patterns classified the SSc subjects by four distinct genetic signatures: diffuse proliferation, inflammatory, limited, and normal-like (42). All three LS subjects' microarray expression aligned with the inflammatory signature on the DEG heat map, expressing primarily T-lymphocyte- and IFNγ-related genes and associating with the early diffuse cutaneous SSc subjects (42).

The other available NGS transcriptional study of LS skin was conducted in pediatric patients but was limited to those with craniofacial scleroderma subtype, termed Parry Romberg Syndrome. RNAseq analyses demonstrated that LS samples (*n* = 16) had increased enrichment groups pertaining to inflammation driven by chemokines/cytokines and interleukins as well as apoptotic signaling which includes genes such as IL24, PROK2, CSF3, RTL1, DPP2, WISP2, and SCARA5 (43).

In our dataset, the comparison of LS to healthy samples clearly identified a distinct transcriptomic difference between disease and healthy skin. Similar enrichment groups related to inflammation as seen in both transcriptional studies performed to date on LS skin were reflected; however, the specific genetic profile of the pediatric LS samples more closely matches the samples within the inflammatory signature that were analyzed with the SSc samples described by Milano et al. Furthermore, within the diverse group of samples included in this study, a distinct subset of patients expressed similar inflammatory genes including interferon-inducible chemokines such as CXCL9, CXCL10, CXCL11, and IFNγ itself. Similar to SSc, subgroupings of LS patients are expected to be present as these inflammatory genes were more highly expressed in LS patients with more inflammatory or active lesions, with associated higher clinical activity scores of mLoSSI and PGA-A. Smaller less distinct subgroupings based on fibrotic and transitioning characteristics are less distinct in this dataset, but clear disease progression in genetic profile is observed.

### Pediatric LS vs. Healthy Control Skin: Inflammation and Fibrosis

A defining gene characteristic in the DEG analysis between all LS patients compared to controls is the inflammatory signaling enrichment groups. A specific interest was taken in the presence of the KRAS signaling pathway, which has previously been defined as a key component of the MAPK/ERK signaling pathway for modulating ERK activity and was highly enriched in the dataset. This pathway plays an important role in cell proliferation, migration, differentiation, and apoptosis and especially T cell infiltration to tissue, including transitioning to T regulatory and TH17 cells (44). The abundance of Tregs and TH17 cells has been contested in recent publications, but the consensus is that both of these cell types are decreased in LS but with an overall increase in interferon expression (all interferon subtypes). Overexpression of the KRAS signaling pathway has been shown to be critical to the development of Th17 cells and the Th17/Treg cell imbalance (45). The KRAS pathway is thought to induce the IRF7 signaling that leads to increased gene expression and induction of key proteins involved in expression and secretion of interferon gene expression inducing the conversion of CD4+-naïve T to Treg cells by upregulating genes that characterize Tregs, including FOXP3 (46–48). In this study, only the KRAS signaling and interferon signaling pathways were observed, including IRF7, with no regulatory component present. The lack of FOXP3 expression observed here and in our prior publication of LS circulating PBMC profile (46) indicates that while the KRAS signaling pathway is inducing interferon-related expression, as seen in the data through high expression of IRF7, IFI27, CCL2, CXCL12, and ARG1, the induction of Th17 and Treg populations is low. Additionally, KRAS and the MAPK/ERK pathway have also been linked to matrix metalloproteinase (MMP) expression (49). MMPs are antifibrotic molecules that induce the degradation of the collagens and other ECM components; unbalanced expressions of MMP and tissue inhibitor of metalloproteinases (TIMP) are involved in the fibrotic process of related autoimmune diseases (50, 51).

### Pediatric Active LS vs. Healthy Control Skin: Inflammation

While signaling and fibrogenic pathways did not enrich in the active LS DEG analysis compared to controls, the overall inflammatory signal dominated the genetic landscape. Enrichment pathways for cytokine and IFNγ signaling predominate, and many genes included in the active LS DEG list correlate with clinical parameters such as disease scoring and lesion number. CXCR3-related ligands, CXCL9, CXCL10, and CXCL11, have repeatedly been presented as active indicators of LS in both protein and transcriptional expressions (11, 38–40). The appearance of these genes as DEGs in active LS expression lists provides further evidence of the importance of these cytokines in LS disease propagation. Transcript staining of these markers in pediatric skin matches immunohistochemistry (IHC) from the skin from adult subjects in the Morphea in Adult and Children (MAC) cohort in which the expressions of TH1 cell markers (CD3, CD4, CXCR3) and CXCL 9 and 10 chemokines were investigated in LS skin (52). A predominate lymphocytic infiltrate was identified in perivascular and periadnexal areas of the superficial and deep dermis of LS subjects, all of which show strong staining with increased percentages of CD3+, CD4+, and CXCR3+ cells (52). The RNAscope findings in our study corroborate the IHC staining by Walker et al., revealing that CXCL9-expressing macrophages reside close to CD4 lymphocytes expressing CXCR3 but neither cell co-expresses the other (52), and supporting our hypothesis that LS macrophage activation mediates IFNγ expression and subsequent T cell CXCR3 receptor binding to overexpress CXCL9 chemokines. Fusiform cells with fibroblast morphology were also observed as CXCL9 expressers in the dermis of LS patients (52). This observation provides some linkage to the idea that fibrosis in LS is inflammatory driven by chemokine fibroblast stimulation and expression.

### Pediatric Inactive LS vs. Healthy Control Skin: Fibrosis

Inactive LS sample DEG enrichment groups predominantly relate to ECM formation and dermal restructuring. Previous studies have identified sub-epithelial thickening and deposition of the extracellular matrix (ECM) as common features of scleroderma skin biopsies (53–55). Fibrogenetic transition states have been implicated in fibrosis, especially in autoimmune-mediated diseases, because of the large role these transition states play in connective tissue composition. Mesenchymal cells such as fibroblasts are the predominant source of many ECM proteins, which is particularly true for fibroblasts that have differentiated into a myofibroblast phenotype (56), i.e., alpha smooth muscle actin (α-SMA)-expressing fibroblasts. Myofibroblasts are known to be the primary source of type I and III collagen in fibrotic lesions, and this is thought to be a consequence of a phenotype differentiation that is dependent on stimulation by TGFβ in many fibrotic diseases (57, 58). In our LS samples, transcription factors that are important in regulating the production of factors that control epithelial–mesenchymal interactions, cellular proliferation, and extracellular matrix production (59, 60) such as WNT, ERK, PI3K- TBX, FOX, RUNX, and SRF were demonstrated to be highly expressed. However, TGFβ is distinctly absent in the LS signature in this dataset. As mentioned, TGFβ is thought to be a key regulator for wound healing and other fibrotic diseases, including SSc, a disease that shares some skin characteristic with LS (61). One of the key factors of this pediatric LS dataset is the lack of TGFβ expression in DEGs between different disease activities and subtypes, which may indicate that TGFβ is not the driving pediatric localized scleroderma fibrosis like it is in SSc skin.

### Pediatric Active LS vs. Inactive LS Skin: Dysregulation

The gene signature of the active samples compared to the inactive samples contains genes related to the general inflammatory response or dermal restructuring as seen in the independent active and inactive comparison to healthy samples, but to a lesser degree of expression change and more truncated gene list. Although the expression is lower, genes related to higher pathway functionality can be seen more clearly than in the other comparisons. The KRAS signaling pathway was among the highest enriched groups with increased expression seen in active samples. This supports our findings from the active samples compared to healthy controls and indicates that this signaling pathway might be directly related to active disease, potentially supporting this pathway as a biomarker of the active disease state in LS. A positive correlation of some of these genes with disease activity scores further supports this relationship.

Of the significantly upregulated pathways associating with disease activity, the JAK/STAT pathways are the most prevalent in DEG comparisons between disease and healthy controls and between active and inactive diseases. These pathways are of clinical interest, as medications inhibiting this pathway exist for autoimmune disease. Inhibitors of these pathways were shown to decrease dermal thickness in a mouse model of LS (62) and improvement symptoms of disease, such as erythema, induration, range of motion, and strength in limited human application. Two of the most used JAK inhibitors, tofacitinib (inhibits Jak1/3) and baricitinib (inhibits Jak1/2), were found to be effective in both human patients and scleroderma mouse models, although the direct mechanism is less clear. The inhibitors might directly inhibit excess collagen production by fibroblasts (62). STAT3 and STAT5 are activated in response to growth, stress, and inflammatory stimuli and are critical for the induction of immune response and pro-proliferative genes (63, 64). Additionally, a direct physical interaction between STAT3 and several NF-κB subunits has been shown to act together to regulate the expression of an overlapping group of target genes (including SERPINE1, BCL3, and BCL2), which result in both transactivation and repression depending on the cellular context (65). While this transcriptomic examination cannot pinpoint direct areas of dysregulation in these pathways, it is highly likely that an active disease in LS is directly linked to those identified which can be examined further in future studies.

## Conclusion

These findings strongly support unique genetic profiles for active and inactive stages of LS and provide areas in which therapeutic intervention could be better targeted. The predominant inflammatory signature in active LS explains why common systemic therapies largely consisting of methotrexate and corticosteroids (66–70) are reasonably effective in the early stages of disease, although they are associated with significant side effects, limiting tolerability and compliance (71–74). The signaling pathways that increased in active disease compared to inactive disease provide further insight to possible disease propagation and potentially pathogenesis, which could help guide current therapies and provide targets for future approaches. The transcriptome classification system in SSc that was determined by Milano et al., has been used more recently to predict patient response to therapy, such as the inflammatory subset showing better response to mycophenolate mofetil and the fibroproliferative group showing better response to stem cell transplant (75, 76). A methodologically similar classification of LS using immunophenotyping of the transcriptome in a higher number of and more diverse group of patients could help to delineate immunological subtypes and determine therapeutic responses to disease.

This study provides the initial foundation demonstrating different immunophenotypes in LS more based on disease activity status rather than disease subtype as a stronger clustering motif. Although all subtypes and activity levels of LS were captured in our study, which is novel to the existing RNA seq literature, sequencing of additional LS sample numbers is underway, which will help to further define these immunophenotypes.

The results of this study provide two novel aspects: (1) an initial dive into the understanding of the pathways promoting and/or sustaining disease in LS by providing initial skin transcriptomic data across disease states and subtypes in LS and (2) demonstration of the utility of RNA seq in paraffinized skin, opening the door to the study of numerous skin conditions using clinically obtained stored specimens, especially helpful in pediatric skin conditions in which a repeat skin biopsy for fresh tissue for research purposes is usually not accepted well by patients and parents.

## Data Availability Statement

The datasets presented in this study can be found online at the NIH GEO repository under GSE 166861.

## Ethics Statement

The studies involving human participants were reviewed and approved by University of Pittsburgh IRB. Written informed consent to participate in this study was provided by the participants' legal guardian/next of kin. Written informed consent was obtained from the minor(s)' legal guardian/next of kin for the publication of any potentially identifiable images or data included in this article.

## Author Contributions

KT obtained funding to perform the study, was in charge of oversight of the study from initiation to completion, reviewed the data and assisted in interpretation, and contributed to manuscript drafting and review. EM contributed to the majority of complex data analyses and summary of results, as well as drafting and editing of the manuscript. JW and RM contributed to additional data analyses and manuscript review. QY, XW, and WC contributed to computational data analysis guidance, experimental design, and manuscript editing. CL and KS contributed to manuscript editing and results presentation. LK provided materials and oversight of RNAScope analyses and manuscript review. All authors contributed to manuscript review and read and approved the final manuscript.

## Conflict of Interest

The authors declare that the research was conducted in the absence of any commercial or financial relationships that could be construed as a potential conflict of interest.

## Abbreviations

LS: localized scleroderma
SSc: systemic sclerosis
RNAseq: RNA sequencing
IFNγ: interferon γ
IRGS: inflammatory response gene signature
CARRA: Childhood Arthritis and Rheumatology Research Alliance
mLoSSI: modified Localized Scleroderma Skin Severity Index
PGA-A: Physician Global Assessment of Activity
TH1: CD4+ IFNγ + T cells
Tregs: FOXP3+ regulatory T cells
TH17: IL17+ T cells
CXCL9: monokine induced by gamma interferon [MIG]
CXCL10: interferon gamma-induced protein 10 [IP-10]
NGS: next-generation sequencing
FF: fresh-frozen
FFPE: formalin-fixed; paraffin-embedded
NRCOS: National Registry for Childhood Onset Scleroderma
GSEA: gene set enrichment
MMP: matrix metalloproteinases
TIMP: tissue inhibitor of metalloproteinases
IHC: immunohistochemistry
MAC: Morphea in Adult and Children
ECM: extracellular matrix
α-SMA: alpha smooth muscle actin
TGFβ: transforming growth factor beta.
